# Optical forces in heat-assisted magnetic recording head-disk interface

**DOI:** 10.1038/s41598-023-35126-3

**Published:** 2023-05-25

**Authors:** Roshan Mathew Tom, Robert Smith, Oscar Ruiz, Qing Dai, David B. Bogy

**Affiliations:** 1 Department of Mechanical Engineering, UC Berkeley, Berkeley, CA 94720 USA; 2 The CTO Office, Western Digital Technologies, San Jose, CA 95119 USA

**Keywords:** Optical physics, Mechanical engineering, Optical physics

## Abstract

A main challenge in Heat-Assisted Magnetic Recording technology is the build-up of contaminants called smear on the near field transducer. In this paper, we investigate the role of optical forces originating from the electric field gradient in the formation of smear. First, based on suitable theoretical approximations, we compare this force with air drag and the thermophoretic force in the head-disk interface for two smear nanoparticle shapes. Then, we evaluate the force field’s sensitivity to the relevant parameter space. We find that the smear nanoparticle’s refractive index, shape, and volume significantly impact the optical force. Further, our simulations reveal that the interface conditions, such as spacing and the presence of other contaminants, also influence the magnitude of the force.

## Introduction

**Figure 1:**
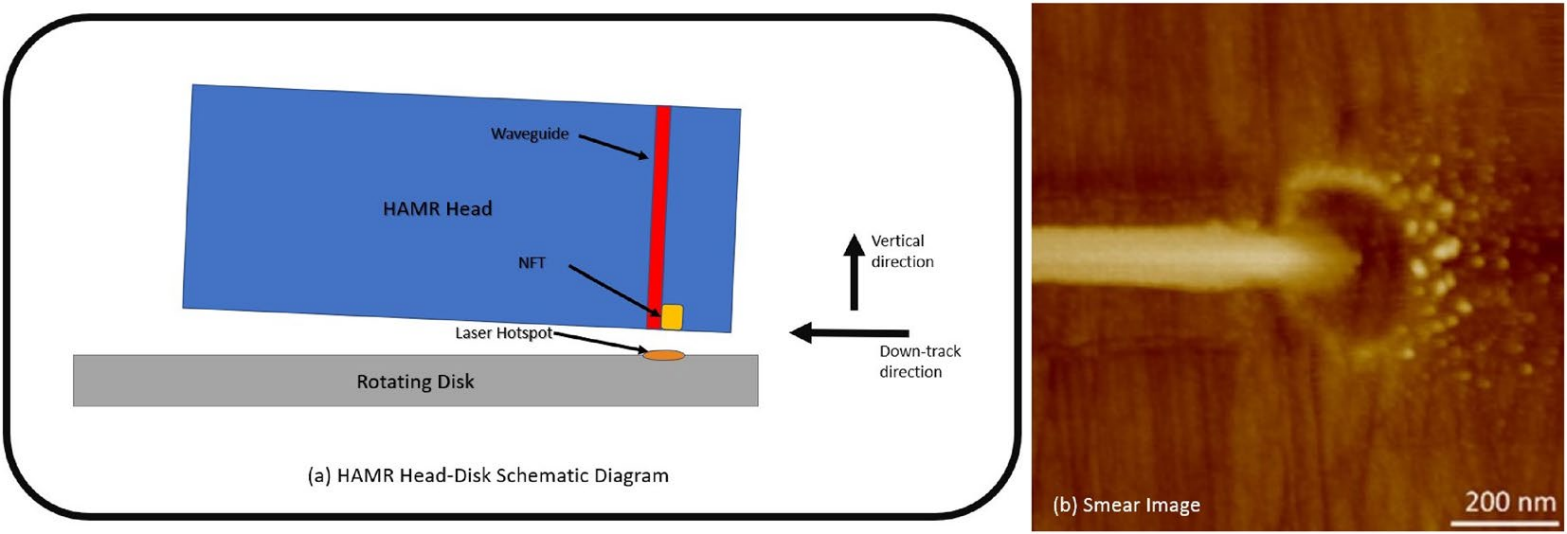
(**a**) Schematic view of the HAMR Head-Disk Assembly (Not to scale). Two directions are also shown relative to the head. The down-track direction is along the circumferential direction on the disk, and the vertical direction is perpendicular to it. The cross-track direction is along the width of the head and into the plane of the schematic (**b**) Experimental image of smear on the head after HAMR writing^5^.

The recording density in conventional disk drive recording technologies is approaching the super-paramagnetic limit, yet, the demand for data storage is higher than ever. Heat-assisted magnetic recording (HAMR) is the leading technology to meet this growing demand^1^. In HAMR, a near-field transducer (NFT) is illuminated with a laser via a waveguide (Fig. 1a). This generates a strong optical near-field at its apex by the excitation of a localized surface plasmon^2^. This surface plasmon is used to heat an FePt-based media to its Curie temperature ($$> 800$$ K) to perform write operations. During this process, the mean head-disk spacing is $$<10$$ nm with pressures in the tens of atmospheres. The temperature field gradient exceeds $$10^9 $$ K/m^3^, and the magnitude of the electric field is about $$7 \times 10^{7} $$ V/m^4^ with a gradient of $$5 \times 10^{16} $$ V/m$$^2$$. These extreme conditions pave the way for contaminations, known as smear, to accumulate on the head^5–7^ (Fig. 1b). A fundamental understanding of smear is critical as it is one key factor limiting the reliability of HAMR drives. Multiple investigations have focused on the temperature-related mechanism driving the formation of smear^8–11^; however, to our knowledge, no study has yet considered the effects of the electric field gradient and its trapping potential.

In his seminal work, Arthur Ashkin^12^ showed that a focused laser beam could trap a microscopic particle due to an optical force. This force forms the basis of optical tweezers. Further, in the last few decades, this theory has been extended to break the diffraction limit of light through plasmonic tweezers^13^, which utilize surface plasmon polaritons (SPP) and localized surface plasmon resonance (LSPR). With the surface plasmon on the NFT and the large electric field gradients across it, the head-disk interface can act as a plasmonic tweezer that traps smear particles. In this study, we look into the effect of this electric field gradient on smear formation. We quantify the optical, drag, and thermophoretic forces using suitable theoretical assumptions. Then, we will compare the magnitude of these forces for a spherical and ellipsoidal nanoparticle to show the relative significance of the optical trap. The results suggest the presence of an optical trap that can influence smear formation. A sensitivity analysis on the relevant parameter space suggests that the smear nanoparticle properties and shape significantly affect the optical force. Additionally, we find that a lower head-disk interface spacing and the presence of foreign contaminants can aid the optical force mechanism of smear formation. Finally, we summarize the results and draw conclusions that will be helpful in the design of the HAMR head-disk interface.

## Derivation of forces

### Optical forces

We begin by assuming that the fundamental smear unit is a particle. This simplifies our analysis as we investigate the force field experienced by the particle at a given position in the head-disk interface. The average electromagnetic force on a particle is determined by the electric and magnetic fields at a closed surface that envelops the particle^14^. The force on the particle can be written as:

1 $$\begin{aligned} \big< \varvec{F} \big>&= \Bigg < \oint _s \varvec{T} \cdot \varvec{n} ds \Bigg > \end{aligned}$$

2 $$\begin{aligned}&= \oint \left\{ \frac{\varepsilon }{2} \text{ Re } \left[ \left( \varvec{E} \cdot \varvec{n} \right) \varvec{E}^*\right] - \frac{\varepsilon }{4} \left( \varvec{E} \cdot \varvec{E}^* \right) \varvec{n} + \frac{\mu }{2} \text{ Re } \left[ \mu \left( \varvec{H} \cdot \varvec{n} \right) \varvec{H}^*\right] - \frac{\mu }{4} \left( \varvec{H} \cdot \varvec{H}^* \right) \varvec{n}\right\} ds \end{aligned}$$

where $\varvec{E}$ and $\varvec{H}$ are the electric and magnetic fields, respectively. $\mu$ and $\varepsilon$ are the relative permeability and the relative permittivity of the surrounding medium, respectively. $\varvec{T}$ is Maxwell’s stress tensor, $\varvec{n}$ is the unit normal perpendicular to the integral area *ds*. The integral is over a surface that encloses the particle. Solving this equation yields two components of force. One is the scattering force, and the other is the optical force^14^. The former points along the in-plane k-vector (that is, along the direction of propagation). It is also known as radiation pressure. In contrast, the optical force is along the electric field gradient vector and is responsible for the optical/plasmonic tweezers effect.

Calculating Maxwell’s stress tensor and the subsequent integration is very difficult to implement and requires lengthy computations. Therefore, we use a suitable approximation to derive a closed-form equation. When the particle size is sufficiently smaller than the wavelength of light, the dipole or Rayleigh approximation is invoked. The particle is approximated as a point dipole acting on an external electric field. The scattering and optical forces under this assumption are given by^14,15^:

3 $$\begin{aligned} \varvec{F}_{scattering}&= \frac{n_m}{c}C_{pr}I {\hat{z}}\end{aligned}$$

4 $$\begin{aligned} \varvec{F}_{optical}&= \frac{1}{2} \text{ Re }\left( \varvec{p} \nabla \varvec{E} \right) \end{aligned}$$

where $n_m$, *I*, and ${\hat{z}}$ are the refractive index of the surrounding medium, intensity of light and the direction of propagation, respectively. $C_{pr}$ is the cross-section of radiation pressure given by,

5 $$\begin{aligned} C_{pr}&= \frac{8}{3} \pi (kr)^4r^2 \left( \frac{\varepsilon - \varepsilon _m}{\varepsilon + 2\varepsilon _m} \right) ^2 \end{aligned}$$

and $\varvec{p}$ is the polarization given by,

6 $$\begin{aligned} \varvec{p}&= 4 \pi \varepsilon _m \varepsilon _0 r^3 \frac{\varepsilon - \varepsilon _m}{\varepsilon + 2\varepsilon _m} \varvec{E} \end{aligned}$$

where *r* is the radius of the particle and $\varepsilon$, $\varepsilon _m$ are the relative permittivity of the particle and the surrounding medium. $\varepsilon _0$ is the vacuum permittivity. *c* and *k* are the speed and wave number of light, respectively. Therefore, we can write the force on the particle as:

7 $$\begin{aligned} \varvec{F}_{scattering}&= \frac{8 \pi n_m k^4 r^6}{3c}\left( \frac{\varepsilon - \varepsilon _m}{\varepsilon + 2\varepsilon _m} \right) ^2 I \end{aligned}$$

8 $$\begin{aligned} \varvec{F}_{optical}&= \frac{2 \pi n_m r^3}{c}\left( \frac{\varepsilon - \varepsilon _m}{\varepsilon + 2\varepsilon _m} \right) \nabla I \end{aligned}$$

where, $I = \frac{1}{2} c \varepsilon _0 n_m | \varvec{E}^2|$ is the intensity of light. Since the optical force is proportional to $r^3$ and the scattering force is proportional to $r^6$, for $r\ll 1$, we have $\varvec{F}_{scattering} \ll \varvec{F}_{optical}$. Thus, we will neglect the scattering effects for the remainder of this report.

The head-disk interface in the vicinity of the NFT spans several tens of nanometers along the surface of the head and less than 10 nm in the vertical direction across the head-disk interface. Thin structures can fit well in this gap. We can approximate the flake-like structure as an ellipsoid with a very high aspect ratio. Gans^16–18^ developed a modified polarization for an ellipsoidal particle given by:

9 $$\begin{aligned} \varvec{p}&= 4 \pi \varepsilon _m \varepsilon _0 r_1 r_2 r_3 \left( \frac{\varepsilon - \varepsilon _m}{3\varepsilon _m + 3L_i(\varepsilon - \varepsilon _m)} \right) \varvec{E} \end{aligned}$$

where $r_i$ and $L_i$ are the radius and a geometric factor in the $i^{\text{ th }}$ direction, respectively. $L_i$ ranges from 0 and $\frac{1}{3}$ and is given by,

10 $$\begin{aligned} L_i&= \frac{r_1r_2r_3}{2} \int _0^\infty \frac{dq}{\left( r_i^2 + q\right) f(q)} \end{aligned}$$

11 $$\begin{aligned} \text{ where, }&f(q) = \sqrt{\left( q + r_1^2 \right) \left( q + r_2^2 \right) \left( q + r_3^2 \right) } \end{aligned}$$

Here, $r_i$ is the radius of the ellipsoid in the respective direction. Using the modified polarization factors, the net optical force is given by,

12 $$\begin{aligned} \varvec{F}_{optical}&= \frac{2 \pi n_m r_1r_2r_3}{c}\left( \frac{\varepsilon - \varepsilon _m}{3\varepsilon _m + 3L_i(\varepsilon - \varepsilon _m)} \right) \nabla I \end{aligned}$$

We call the term in the parenthesis the enhancement factor $\left( E_f \right)$ as it indicates the field enhancement that a particle induces around itself. The denominator in this term indicates the possibility of a singularity when the geometric factor and permittivity satisfy the Fröhlich condition, given by:

13 $$\begin{aligned} 3\varepsilon _m + 3L_i(\varepsilon - \varepsilon _m)&= 0 \end{aligned}$$

14 $$\begin{aligned} \Rightarrow \varepsilon = \varepsilon _m \left( 1-\frac{1}{L_i}\right) \end{aligned}$$

As the permittivity of the particle approaches the value given by Eq. (14), the force experienced by the particle drastically increases. Since $L_i$ is always less than 1, the condition is met only when the real part of the permittivity is negative. In such materials, the imaginary component of the permittivity is non-zero; thus, the force does not reach a singularity. Nevertheless, this condition is of interest because when the real part does satisfy the Fröhlich condition, the optical force reaches its peak value. The relevance of this condition will be explored when we study the material dependence of the optical force.

### Thermophoretic force

Thermophoresis is the collective action of Brownian motion of the air particles due to a temperature gradient. Due to the uneven temperature on the two ends of the particle, a net force is generated that is directed toward the cooler side^19^. Since the head-disk interface has a temperature gradient from the hot disk to the relatively cooler head, a thermophoretic force directed towards the NFT may act on a smear particle.

Although the air pressure in the head-disk interface is in the continuum limit, the mean free path of a gas molecule is much larger than the characteristic length of the head-disk space, so a free-molecular gas limit is more appropriate. The thermophoretic force, in this case, was developed by Torczynski and reported in Gallis^20^.

15 $$\begin{aligned} F_T&= - \left( \frac{3}{2} \pi r^2 nk_B\sqrt{(T_CT_H)} \right) \left( \frac{T_H^{1/2} - T_C^{1/2}}{T_H^{1/2} + T_C^{1/2}} \right) \end{aligned}$$

Where *r*, *n*, $k_B$ are the sphere’s radius, the number density of the gas, and the Boltzmann constant. $T_H$ and $T_C$ are the temperature in the hot and cold end, respectively. Since this equation was developed for a sphere and not an ellipsoid, we will limit the use of the formula to the spherical nanoparticle.

### Drag force

In the case of drag force, the forces can be thought of as either the pressure drag or the skin friction drag. In the case of the pressure drag, Epstein formulated the drag force in the free molecular regime as^21^:

16 $$\begin{aligned} F&= \frac{2}{3} r^2 \rho _g \sqrt{\frac{2 \pi k_BT}{m_g}} \left( 1 + \frac{\pi \alpha }{8}\right) \end{aligned}$$

where $\alpha$ is a factor depending on the collision of the gas particle with the nanoparticle’s surface. In the case of very small spheres, $\alpha$ is close to 1. In the case of an ellipsoidal structure with a high aspect ratio, skin-friction drag force dominates. The skin-friction component can be approximated by the shear force experienced by the head. The total shear force on the head is calculated using an air-bearing simulation. If the total shear force is $F_{s,total}$, then

17 $$\begin{aligned} F_{s,ellipsoid}&= 2F_{s,total} \frac{A_{ellipsoid}}{A_{head}} \end{aligned}$$

where $A_{ellipsoid}$ and $A_{head}$ are the projected surface areas of the ellipsoid and the head, respectively.

Apart from the thermophoretic and drag forces, many other forces exist in a typical HAMR head-disk interface. One important force is the van der Waals force that attracts the particles to the head or the disk. However, given the high temperature and complex materials in the head and the disk, it is difficult to accurately compute van der Waals forces. Therefore this study will restrict comparing the optical force to the thermophoretic and drag force.

## Results

### Simulation assumptions

**Figure 2:**
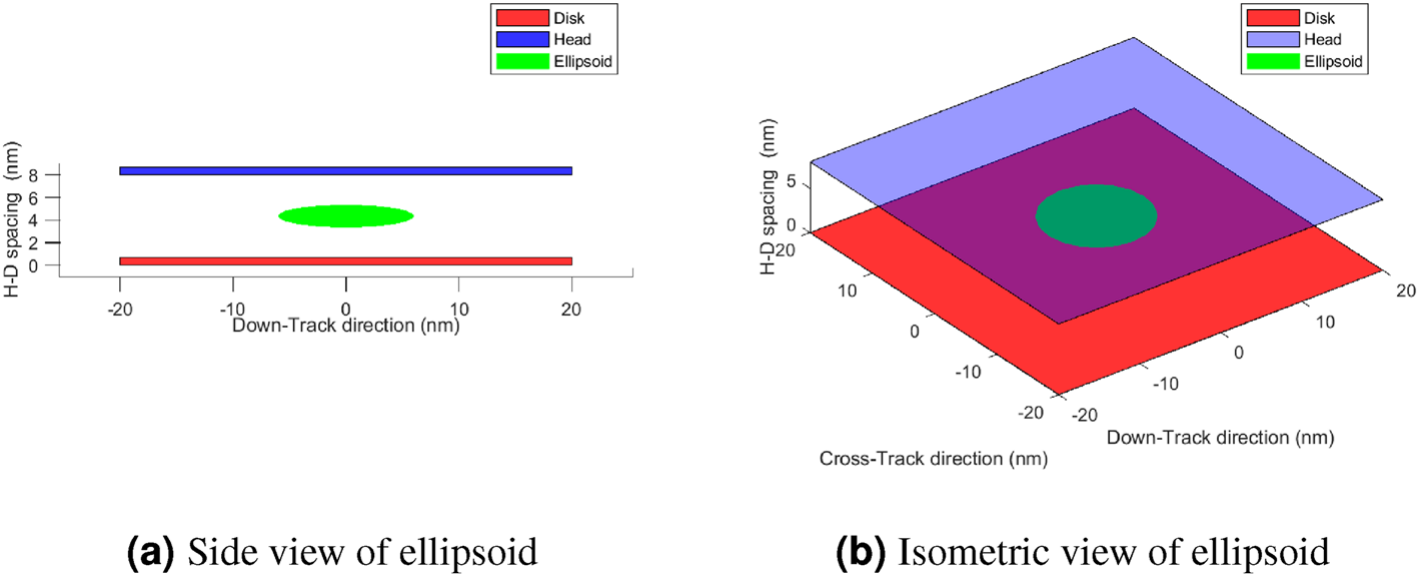
Illustration of the Ellipsoid: The blue and red planes represent the top and bottom surfaces of the disk and head, respectively. The green disk at the center is the ellipsoidal smear nanoparticle. (**a**) The side view of the Ellipsoid, (**b**) The isometric view.

The first set of simulations starts with a clean air-filled head-disk interface with a spacing of 8 nm. The clean air was used to understand the origin of smear accumulation. The air density is calculated at a pressure of 25 atm. The representative mass of each air molecule is $4.3 \times 10^{-26}$kg. We simulate two particles to compare the forces. One is a spherical nanoparticle with a radius of 1 nm, and the other is an ellipsoid with radii 6nm in the in-plane direction and 1 nm in the vertical direction (see Fig. 2). The particles have a refractive index of 1.53 at 830 nm light and a density of $2650 \; \text {kg}/\text {m}^3$, corresponding to a SiO$_2$ particle. The forces are calculated in three directions. First, the down-track direction that is along the length of the head and parallel to the write-track. Second, the cross-track direction that is perpendicular to the write-track and along the width of the head. The third is the vertical direction that is perpendicular to the recording medium and the head air-bearing surface. The down-track and vertical directions are shown in Fig. 1a. The forces were calculated at each point in the plane, and the resulting forces were plotted as a heat map. Each point in this heat map shows the magnitude of the force in the direction that the map represents.

The electric field at the interface of the HAMR head-disk assembly is found by solving Maxwell’s equations using a frequency domain finite element method simulation of an internal HAMR head-disk assembly in CST Microwave Studio^10^. A steady-state solver is then used to calculate the temperature field. Due to the proprietary nature of the design, we are unable to publish the electric and temperature fields in the public domain; however, they have been used as the basis for calculating the optical forces presented in this report.

### Spherical particle

**Figure 3:**
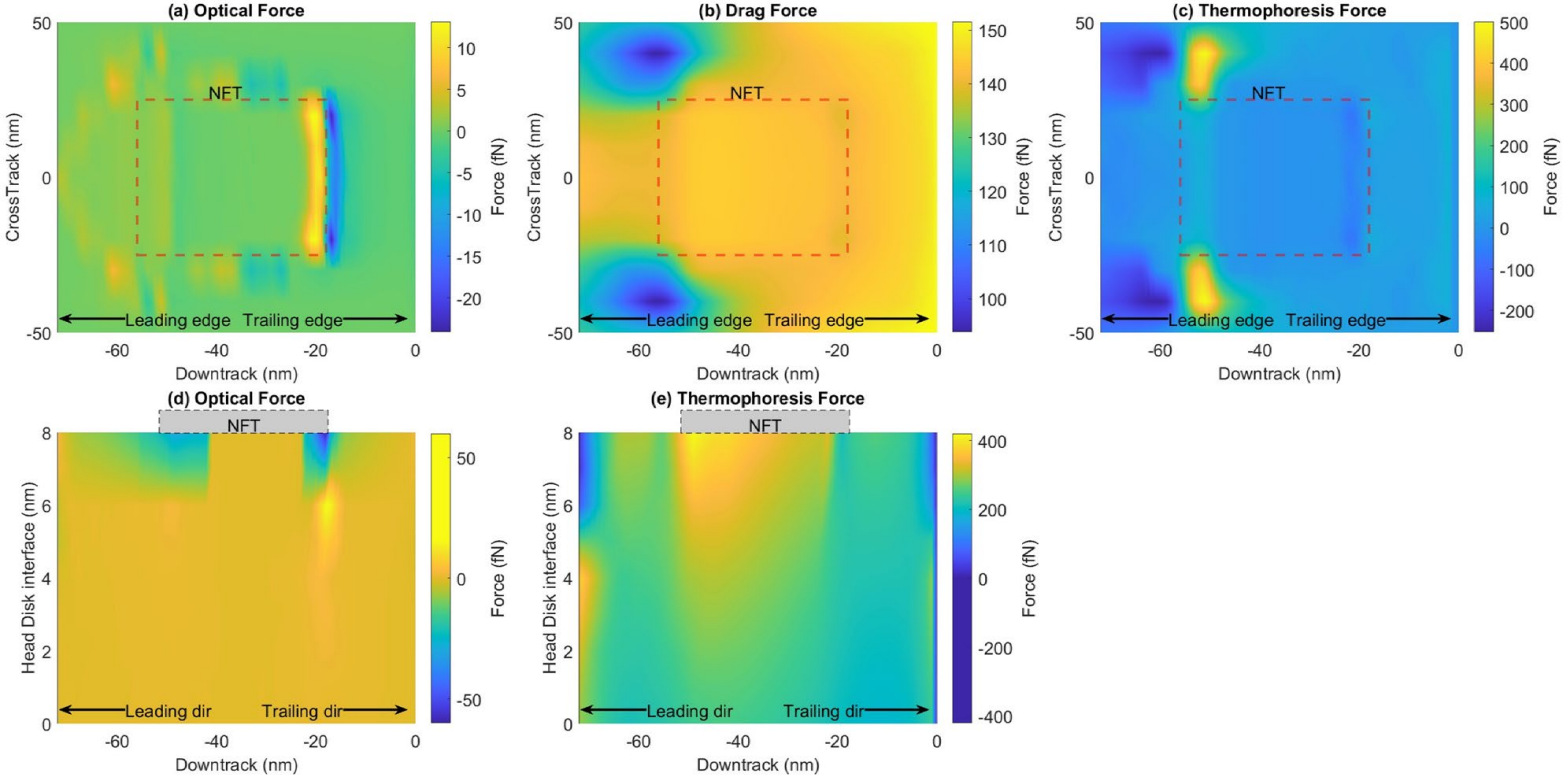
Comparison of forces on a spherical particle: (**a**) Optical force in the down-track direction on a plane 1nm below the head, (**b**) the drag force in the down-track direction, (**c**) the thermophoretic force in the down-track direction, (**d**) the optical force in the vertical direction, (**e**) the thermophoretic force in the vertical direction.

In the case of the spherical particle, the drag, thermophoretic, and optical forces are shown in Fig. 3. The magnitude of the optical force is about 25 fN in the down-track direction and 60 fN in the vertical direction. A stagnation point is observed near the trailing end of the Near Field Transducer (NFT). This force would cause the nanoparticle to stay near the NFT. Some of the features of the forces in the down-track direction are asymmetrical as the underlying electric field gradient is asymmetric. With the forces in the vertical direction (Fig. 3d), we see a positive force pulling the particle towards the NFT (away from the disk). Nevertheless, the forces are smaller compared to the drag and thermophoretic forces. They indicate that the optical forces on the spherical SiO$_2$ particles would not overcome the forces, pushing the particle downstream of the NFT.

### Ellipsoidal particle

**Figure 4:**
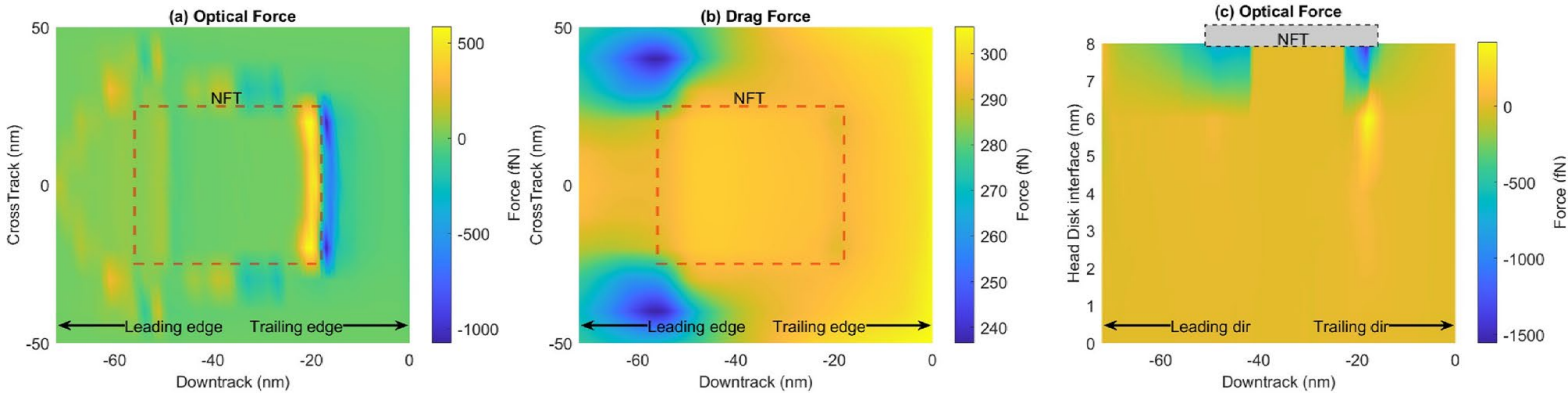
Comparison of forces on an ellipsoidal particle: (**a**) Optical force in the down-track direction, (**b**) the thermophoretic force in the down-track direction, (**c**) Optical force in the vertical direction.

In the case of the ellipsoidal particle, the optical forces and drag force are shown in Fig. 4. The overall behavior and pattern of the optical forces are similar to that of the spherical case. However, it is worth noting that for an ellipsoidal shape, the force acting on the particle is an order of magnitude larger than the spherical-shaped particle. The optical force on the ellipsoidal particle is 44x larger, whereas its drag force is 2x larger. This is because the optical force is directly proportional to the volume, and the ellipsoidal particle achieves a much larger volume in the thin interface than the sphere. The drag force on the ellipsoidal shape does not increase as dramatically with volume since the ellipsoid behaves as a streamlined body. Thus ellipsoidal or slender-shaped SiO$_2$ particles would experience significant optical forces. The opposing forces near the trailing edge of the NFT induce a potential well that traps a smear particle against the opposing drag force.

In the vertical direction (Fig. 4c), the forces are negligible near the disk. However, as the particle approaches the NFT, the optical force gradually increases. Specifically, large forces are seen near the leading and trailing edge of the NFT. This occurs because the surface plasmon generated by the laser has its peak intensity at these locations. The opposing forces create an entrapment zone such that a smear particle flying in the vicinity of the NFT is captured by it. Combined with the forces seen in the down-track direction and cross-track direction, the optical trap is formed that can confine smear particles in the head-disk interface. This confinement can initiate a smear buildup in the head.

## Sensitivity analysis of optical force

### Material dependence

**Figure 5:**
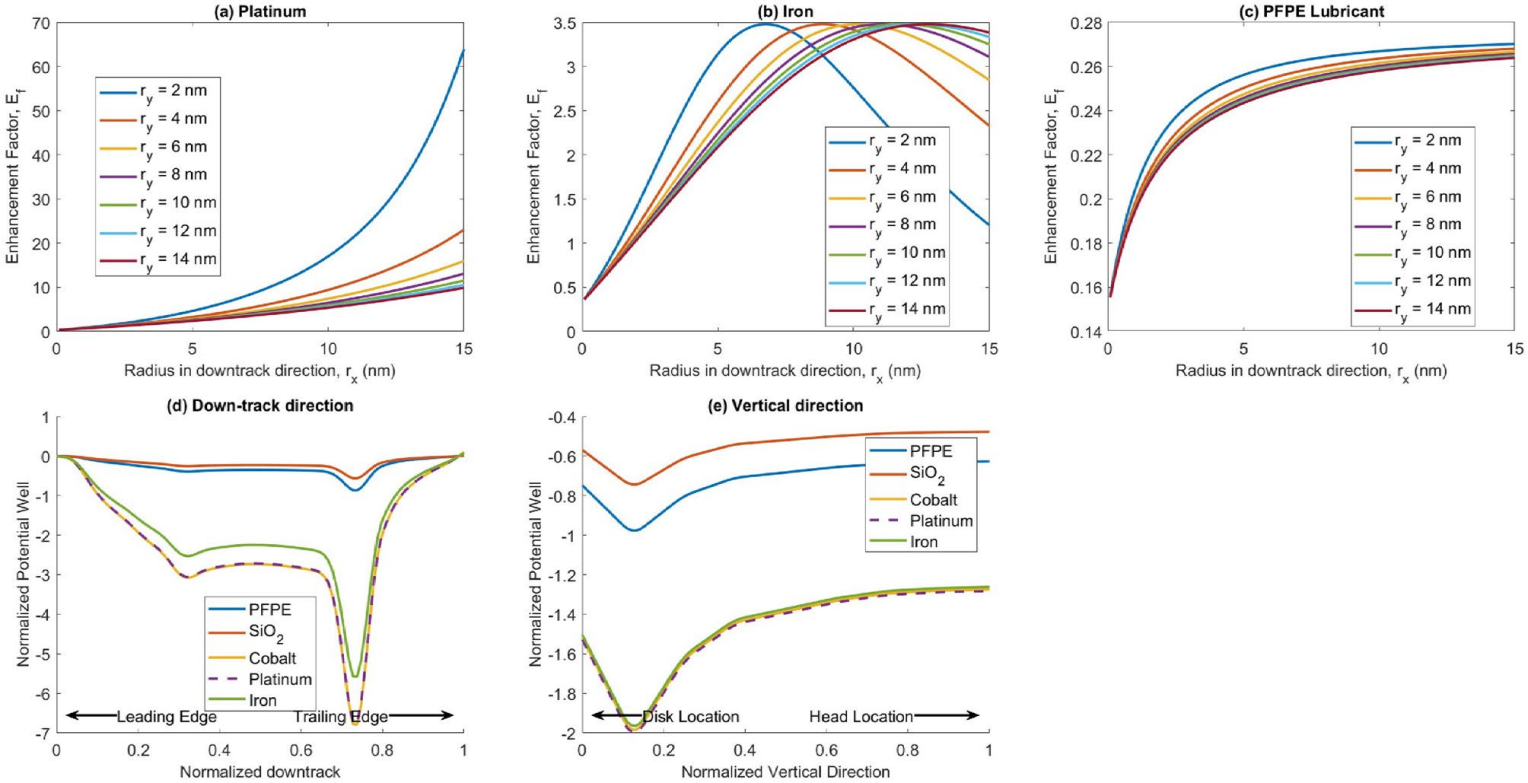
(**a**–**c**) Field Enhancement for Platinum, Iron, and PFPE Lubricant. $r_x$ is the radius in the down-track direction, $r_y$ is the radius in the cross-track direction and $r_z$, the radius in the vertical direction is fixed at 1 nm. (**d**) The normalized potential well in the down-track direction on a plane 1nm below the head for various materials (e) The normalized potential well in the vertical direction for various materials.

In addition to SiO$_2$, smear can originate from a complex combination of materials, including HAMR disk metals such as Iron, Platinum, and Cobalt, and dielectrics such as PFPE Lubricant^22,23^. In this sub-section, we vary the smear material to determine the impact on the optical force. The materials’ permittivities primarily drive the variation in optical forces. The component containing the relative permittivity is the field enhancement factor $E_f$. Using the relative permittivity values (square of the refractive index) found in the literature for different particles in Table 1, we calculated the optical force along the down-track, cross-track, and vertical directions. The potential well obtained from these graphs is plotted in Fig. 5. The results indicate that the metals generally experience much larger optical forces than dielectrics. Further, even among the metals which were evaluated, platinum and cobalt have the deepest potential well. Among the dielectrics, silica particles have larger optical forces than PFPE lube. These differences can be attributed to the different enhancement factors associated with the different materials.

We plot the enhancement factor for these materials as a function of the aspect ratio in Fig. 5a–c. In these plots, the radius in the vertical direction, $r_z$ is set at 1 nm. In the case of Iron, when $r_y = 1$nm and $r_x = 6.76$ nm, the geometric factor, $L_i = 0.0578$. The permittivities of iron and air are $2.9425 + 3.4115i$ and 1, respectively. When we insert these values in the Frölich equation, we get $E_f = 3.4776$, which is the point where the enhancement factor is at its peak. This peak is because the real part of its permittivity meets the Frölich condition. As mentioned earlier, the imaginary term of the relative permittivity prevents a singularity. Similar behavior is seen for both Cobalt and Platinum. The peak dimensions are plotted in Table 2. For platinum, the enhancement factor rises rapidly to values exceeding iron by an order of magnitude. However, these values are reached only at extremely high aspect ratios. At those dimensions, the particle can no longer be approximated as a point dipole with a uniform electric field gradient across it. The equations developed earlier would, therefore, no longer apply.

In the case of PFPE (Fig. 5c), the enhancement factor grows until it reaches a steady peak value. Any increase in the aspect ratio has negligible effects on the enhancement factor. A similar result can be observed for SiO$_2$ as well. For PFPE and SiO$_2$, the peak $E_f$ values are 0.27 and 0.44, respectively. These values are less than 1, so we call them attenuation rather than enhancement. This result is consistent with the lower potential wells seen in Fig. 5.

**Table 1:** Refractive indices of different materials at $\lambda = 830$ nm.

Material	Refractive index $\left( \sqrt{\varepsilon } \right)$
Iron (Fe)^24^	$2.9425 + 3.4115i$
Cobalt (Co)^24^	$2.5452 + 4.9155i$
Platinum (Pt)^25^	$0.601383 + 8.4208i$
PFPE lube	1.35
Silica	1.53

**Table 2:** $r_x$ and $r_y$ combinations when the peak enhancement factors occur for different elements. $r_z$ is fixed at 1 nm.

$r_y$ (nm)	$r_x$ (nm)		
	Iron	Cobalt	Platinum
2	6.76	8.09	16.45
6	10.16	12.31	26.02
10	11.76	14.38	31.32
$E_f$ (Peak)	3.4776	6.49	85.92

### Effect of head disk spacing

**Figure 6:**
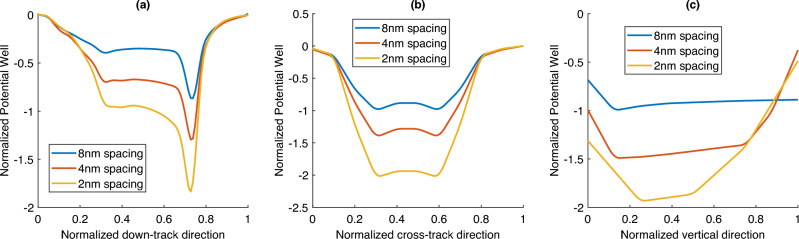
Optical forces for varying spacing: (**a**) down-track direction (calculated on a plane 1 nm below the head), (**b**) cross-track direction (calculated on a plane 1 nm below the head), and (**c**) vertical direction.

The head-disk spacing in HAMR varies depending on a variety of factors. Here, we examine how the optical forces change with varying head-disk spacing. As in the previous sections, an electromagnetic analysis is done for head-disk spacings of 8 nm, 4 nm, and 2 nm. Optical forces on an ellipsoidal SiO$_2$ particle were then calculated and analyzed. The normalized potential wells from these cases are shown in Fig. 6. All three plots show that decreasing the spacing results in an increase in the optical force. Thus, in general, operating at higher spacing may potentially reduce optically-induced smear collection. Further, the increase in the potential depth as we go from 8 nm to 4 nm and that from 4nm to 2nm is similar. This suggests that the rate at which the optical force decrease also decreases at higher spacing. Thus, there is a limit beyond which any increase in spacing will not result in a meaningful drop in the optical force.

### Effect of contamination

Our analysis assumed a clean air interface with no contamination in all previous simulations. However, in operating conditions, the head-disk interface contains many contaminants. In this sub-section, we analyze the force experienced by an ellipsoidal nanoparticle of smear in an environment where other smear contaminants are already present. We consider two kinds of contaminants. Since organic materials, such as the lubricant that coats the disk, are plentiful at the interface, our first contaminant will be a layer of organic smear on the head. Second, as we have shown, platinum exhibits a significant enhancement factor and has the potential to have a considerable influence on the forces experienced by a secondary nanoparticle. So we will introduce a nano-sized metallic body made of platinum in the interface and analyze the results.

#### Layer of organic smear

**Figure 7:**
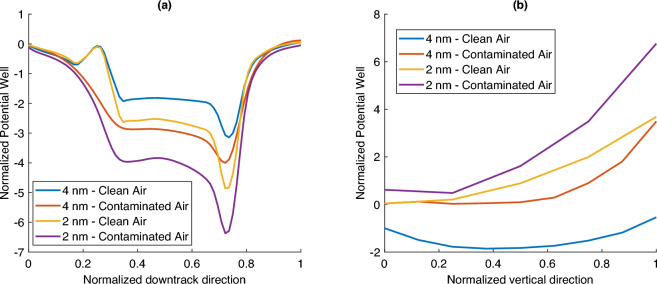
Normalized potential well along different directions for varying spacing and contamination rates: (**a**) down-track direction, and (**b**) vertical direction.

In this case, we model the head-disk interface in two layers. The first layer attached to the disk was clean and free from contaminants, and the second layer attached to the head was made entirely of organic smear. By varying thicknesses of the two layers, this configuration resembles the growth of the smear on the head surface over time. Taking the refractive index of the organic layer to be 1.3, we calculated and analyzed the optical force in the down-track and vertical direction for each case. The forces are normalized using the peak force, and the potential wells obtained are shown in Fig. 7. The first case is at a total spacing of 4 nm without any smear, the second case when the total spacing is 4 nm with 2 nm each of air and smear, and the third case is at a total spacing of 2 nm without any smear, and the fourth case is with a total spacing of 2 nm with 1nm of air and smear.

The rise in force when we introduce the smear for the 2 nm and 4 nm cases is by a factor of 1.5. Therefore, if we fix the spacing while allowing the smear to build up on the head, the optical forces experienced by a smear nanoparticle in the layer of air increase. Further, the potential well in the second and third cases follow a similar path at critical locations. In both these cases, a 2 nm layer of air is present. This shows that the thickness of the layer of air determines the optical force on a smear nanoparticle. When the smear accumulates on the head’s surface, the effective depth of the clean air reduces. Thus, the interface behaves as if the head-disk spacing is reduced when we estimate the optical force. These results highlight the importance of keeping the head-disk interface free of organic smear layers. Otherwise, the optical force can promote additional smear growth.

#### Presence of a metallic body

**Figure 8:**
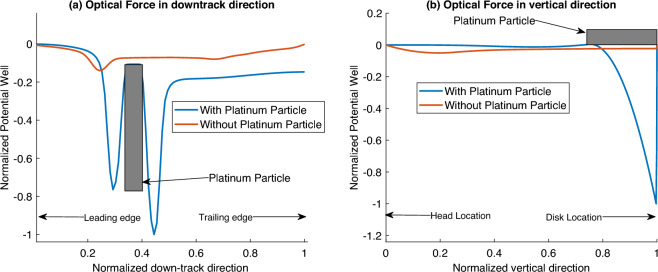
Normalized potential well along different directions for the case with and without a platinum particle contamination: (**a**) down-track direction, and (**b**) vertical direction.

In this case, we introduce a cylindrical object made of platinum into a clean 8 nm thick head-disk interface. The cylinder has a radius of 10 nm and a length of 2.2 nm. The platinum particle was placed on the disk. We then calculate the optical force and examine the net force field generated by the primary interface particle on a secondary nanoparticle. The force field is normalized with the force when the object is not present. The subsequent potential well in the down-track and vertical direction is shown in Fig. 8.

We found that the potential well’s depth in the presence of the particle is about 8 times greater in the down-track direction and 20 times greater in the vertical direction. The peak drop in the potential well is found to be near the surface of the cylinder. The sharp drop is due to a secondary surface plasmon being generated at the metal-air interface of the object. This secondary plasmon induces a large electric field gradient capable of trapping other smear nanoparticles. Therefore, smear particles in the vicinity of the metallic object are drawn towards it rather than the NFT. This attraction and subsequent adhesion to the metallic object could cause it to grow in size. Effectively, the original particle and those surrounding it behave as a composite object with an arbitrary shape. This composite object now has a much larger volume and, consequently, a much larger optical force acting on it induced by the electric field of the NFT.

## Conclusion & scope for future work

This paper quantifies the optical force on a smear nanoparticle in the Head-Disk interface. Further investigation on the relevant parameter space revealed the conditions where the optical force can have appreciable effects on smear formation. These factors can be categorized as smear and interface parameters.

The key smear parameters are the shape, material, and volume of the smear nanoparticle. Increasing the volume of the particle results in larger forces. In the film-like head-disk spacing, the increase in volume is achieved by considering a disk/ellipsoidal-shaped smear flake. An ellipsoidal shape combined with appropriate permittivity in metals also allows for the Fröhlich condition to be satisfied, which enhances the effect by 3 to 6 times. Thus, metals experience a much higher optical force, even when present in relatively small quantities. Dielectrics such as Silica and PFPE Lube do not experience field enhancement but rather see an attenuation. Nevertheless, in large quantities, dielectrics can experience significant optical forces.

The interface parameters are the head-disk spacing and the presence of other contaminants. Lowering the head-disk spacing increases the optical force on a smear nanoparticle. Additionally, the presence of contaminants like an existing smear layer and a metallic particle can increase the optical force experienced by a secondary smear particle. Metallic contaminants have the greatest influence on optical forces. The force increase is more than an order of magnitude larger than the case without the particle. This is due to the formation of a secondary surface propagating plasmon at the metallic particle-to-air interface.

Since optical forces are dependent on the magnitude of the electric field gradient, a change in the NFT design may result in different optical forces. Nevertheless, when an NFT generates a surface plasmon, optical forces will be present near it. The magnitude of the force depends on the magnitude of the electric field gradient. Further studies can be done to understand how optical forces depend on NFT designs. Future investigations will also explore more complex optical force models with additional considerations. One crucial consideration is the permittivity of the different materials. We have assumed that the smear nanoparticle has the same permittivity as its bulk counterpart. However, the extremely small size of the particle would alter the permittivity. The effect of the modified permittivity values would be of interest. Another area to investigate would be to look beyond the Rayleigh approximation to calculate the exact force on a smear nanoparticle. Maxwell’s equations can be used to calculate the scattered electric and magnetic fields. These fields can then be used to calculate the exact optical force using Maxwell’s stress tensor method (Eq. 2). The results can then be used to compare the validity of the Rayleigh approximation used in this report.

## Data Availability

The datasets used for the current study may be available from the corresponding author on reasonable request.

## References

[1] Kryder M (2008). Heat assisted magnetic recording. Proc. IEEE.

[2] Matsumoto T, Akagi F, Mochizuki M, Miyamoto H, Stipe B (2012). Integrated head design using a nanobeak antenna for thermally assisted magnetic recording. Opt. Express.

[3] Saunders DA, Hohlfeld J, Zheng X, Rausch T, Rea C (2017). HAMR thermal gradient measurements and analysis. IEEE Trans. Magn..

[4] Xu B (2013). HAMR media design in optical and thermal aspects. IEEE Trans. Magn..

[5] Xiong S (2017). Material transfer inside head disk interface for heat assisted magnetic recording. Tribol. Lett..

[6] Kiely JD, Jones PM, Hoehn J (2018). Materials challenges for the heat-assisted magnetic recording head–disk interface. MRS Bull..

[7] Cheng Q, Bogy DB (2022). Experimental study of smear formation and removal in heat-assisted magnetic recording. Tribol. Int..

[8] Sakhalkar SV, Bogy DB (2018). Effect of rheology and slip on lubricant deformation and disk-to-head transfer during heat-assisted magnetic recording (HAMR). Tribol. Lett..

[9] Sakhalkar SV, Bogy DB (2019). Viscoelastic lubricant deformation and disk-to-head transfer during heat-assisted magnetic recording. IEEE Trans. Magn..

[10] Smith, R., Rajauria, S., Brockie, R., Schreck, E. & Dai, Q. Opto-thermal simulation of metallic smear’s impact on hamr technology. In *2021 IEEE 32nd Magnetic Recording Conference (TMRC)*, 1–2, 10.1109/TMRC53175.2021.9605106 (2021).

[11] Tani H, Lu R, Koganezawa S, Tagawa N (2020). Investigation of mechanism of smear formation from diamond-like carbon films on heating. Microsyst. Technol..

[12] Ashkin A (1970). Acceleration and trapping of particles by radiation pressure. Phys. Rev. Lett..

[13] Righini M, Volpe G, Girard C, Petrov D, Quidant R (2008). Surface plasmon optical tweezers: Tunable optical manipulation in the femtonewton range. Phys. Rev. Lett..

[14] Zhang Y (2021). Plasmonic tweezers: For nanoscale optical trapping and beyond. Light Sci. Appl..

[15] Harada Y, Asakura T (1996). Radiation forces on a dielectric sphere in the Rayleigh scattering regime. Opt. Commun..

[16] Gans R (1912). Über die form ultramikroskopischer goldteilchen. Ann. Phys..

[17] Kreibig U, Vollmer M (1995). Optical Properties of Metal Clusters.

[18] Maier SA (2007). Plasmonics: Fundamentals and Applications.

[19] Gallis MA, Rader DJ, Torczynski JR (2004). A generalized approximation for the thermophoretic force on a free-molecular particle. Aerosol. Sci. Technol..

[20] Gallis, M., Rader, D. & Torczynski, J. Dsmc simulations of the thermophoretic force on a spherical macroscopic particle. In *35th AIAA Thermophysics Conference*, 10.2514/6.2001-2890 (American Institute of Aeronautics and Astronautics, 2001).

[21] Epstein PS (1924). On the resistance experienced by spheres in their motion through gases. Phys. Rev..

[22] Kiely JD (2017). Write-induced head contamination in heat-assisted magnetic recording. IEEE Trans. Magn..

[23] Xiong S, Smith R, Schreck E, Dai Q (2021). Experimental study of material pick up on heat-assisted magnetic recording (HAMR) heads. Tribol. Lett..

[24] Johnson PB, Christy RW (1974). Optical constants of transition metals: Ti, V, Cr, Mn, Fe, Co, Ni, and Pd. Phys. Rev. B.

[25] Werner WSM, Glantschnig K, Ambrosch-Draxl C (2009). Optical constants and inelastic electron-scattering data for 17 elemental metals. J. Phys. Chem. Ref. Data.

